# Modified eggshell procedure via posterior approach for sclerosing thoracic disc herniation: a preliminary study

**DOI:** 10.1186/s13018-016-0438-2

**Published:** 2016-09-20

**Authors:** Si-Dong Yang, Qian Chen, Sheng-Hua Ning, Wen-Yuan Ding, Da-Long Yang

**Affiliations:** 1 Department of Spinal Surgery, The Third Hospital of Hebei Medical University, 139 Ziqiang Road, Shijiazhuang, 050051 China; 2 Department of Orthopaedic Surgery, Zhongshan Hospital, Fudan University, 180 Fenglin Road, Shanghai, 200032 China; 3 Hebei Provincial Key Laboratory of Orthopaedic Biomechanics, 139 Ziqiang Road, Shijiazhuang, 050051 China

**Keywords:** Thoracic disc herniation, Eggshell technique, Modified eggshell procedure, Technical modification

## Abstract

**Background:**

Clinically, sclerosing thoracic disc herniation is a disease with high surgical risk and various complications. Eggshell procedure is a surgical method used by surgeons to treat sclerosing thoracic disc herniation. The aim of this study was to report a modified eggshell procedure to treat sclerosing thoracic disc herniation.

**Methods:**

Medical records of 25 patients with sclerosing thoracic disc herniation were collected between 2007/01 and 2010/08, including 14 males and 11 females, with an average age of 51.7 years old. Modified eggshell procedure was performed to treat the patients with sclerosing thoracic disc herniation. All patients were followed up. Japanese Orthopaedic Association (JOA) score was used to evaluate the clinical outcomes.

**Results:**

All operations were performed successfully with complication rate of 12 %. There were 2 cases of dural laceration and 1 subdural hematoma. All included patients were followed up for at least 5 years, with the median of 6 years. JOA score of preoperation was 5 (IQR = 1) while it was 8 (IQR = 2) at final follow-up, with significant difference (Mann-Whitney *U* test, *Z* = −4.891, *P* < 0.001). The improvement rate of neurological status was 51.5 ± 23.1 %. According to the classification of improvement rate, there were 15 cases at good level, 8 cases at moderate level, and 2 cases without any improvement.

**Conclusions:**

Modified eggshell procedure is a safe and effective surgical method when performed to treat sclerosing thoracic disc herniation in the clinical practice.

## Background

Due to its special anatomical structure of thoracic vertebra, the incidence of thoracic disc herniation is low [1]. The herniated disc is often associated with calcification and a series of hardening performance, including ossification of posterior longitudinal ligament, the posterior marginal osteophyte formation, and so on [2]. It is known as hardening thoracic disc herniation, which is a kind of disease with high surgical risk and various complications [3, 4]. The clinical manifestations of hardening thoracic disc herniation are often severe [4, 5]. The herniated and hardening part usually deepens into the ventral spinal dural with adhesions, even causing mechanical compression on thoracic spinal cord and reducing blood circulation of spinal cord by affecting the anterior spinal central artery. Once the symptomatic thoracic disc herniation is confirmed, the patient had better receive surgical treatment as soon as possible [6]. The surgery indications [7, 8] include progressive spinal cord damage, lower limb weakness or paralysis, bowel and bladder dysfunction, segmental sensory disturbances, and persistent radiating pain. There are several surgical methods [5] to treat thoracic disc herniation. Surgical procedures of decompression via posterior laminectomy and discectomy are relatively simple. However, even with simple procedures, intraoperative traction to the affected spinal cord is still easy to lead to spinal cord injury. Thus, these surgical methods are not satisfying.

To our knowledge, “eggshell procedure” was firstly used for anterior decompression of spinal fractures via a posterior way and then used widely [9]. Firstly termed by Heinig in 1985, eggshell procedure is that vertebral body is firstly hollowed out via vertebral pedicle, and then the protruding fracture block is rammed into the hollowed vertebral body. In this study, we modified this procedure and applied it to the surgical treatment of sclerosing thoracic disc herniation. In a clinical practice, it proved to be safe and effective. Hence, we tried to report this modified procedure herein.

## Methods

### Ethics statement

This retrospective study has been approved by Ethics Committee of the Third Hospital of Hebei Medical University. The approval number is K2015-019-02.

### Patients

Medical records of 25 patients with sclerosing thoracic disc herniation were collected between 2007/01 and 2010/08, including 14 males and 11 females, with an average age of 51.7 years old (ranging from 32 to 70 years). Of them, lower limb numbness appeared in four cases, and the disease progression was rapid. The rest patients showed a progressive development. Modified eggshell procedure was performed to all patients to treat sclerosing thoracic disc herniation. All patients were followed up on regular. Japanese Orthopaedic Association (JOA) score was used to evaluate clinical outcomes.

### Clinical manifestations

All patients presented varying degrees of progressive numbness, weakness and stiffness at lower limbs, and they felt as if “step on the cotton” when walking. Ten cases of them presented belted sense in chest and abdomen. Fourteen cases presented as upper motor neuron damage, including high lower extremity muscle tone, tendon hyperreflexia, and positive pathological syndrome. Eight cases simultaneously presented upper and lower motor neuron damage, including normal or high muscle tone, tendon hyporeflexia and positive pathological syndrome, and weakened muscle strength. Seven cases showed bowel and bladder dysfunction.

### Imaging examination

All of the patients received X-ray, computed tomography (CT) scan, and magnetic resonance imaging (MRI) scan before surgical treatment, all showing single disc herniation (4 cases at T8–9 level, 10 cases at T9–10 level, 7 cases at T10–11 level, 4 cases at T11–12 level). As for the herniation type, there were 18 cases with central herniation and 7 cases with paracentral herniation. The X-ray results showed that there was hyperostosis in the posterior margin of the vertebral body, and the intervertebral space was narrow in all of the patients. The adjacent vertebral bodies were irregular in the lesion site with 12 cases, including wedge-shaped deformation (7 cases), biconcave change (2 cases), and the upper and lower edge hardening (3 cases). The results of CT scan showed that there was disc calcification in the affected site, including 5 cases with yellow ligament ossification. The results of MRI scan showed that there were different degrees of spinal cord compression and deformation in the affected area.

There were abnormally high signal changes of T2-weighted imaging (T2WI) in the spinal cord with 19 cases. The nucleus pulposus protrusion presented as mixer of high signal and low signal on T1-weighted imaging (T1WI) and T2WI images, and most of the part was low signal.

### Surgical technique and postoperative treatment

**Fig. 1:**
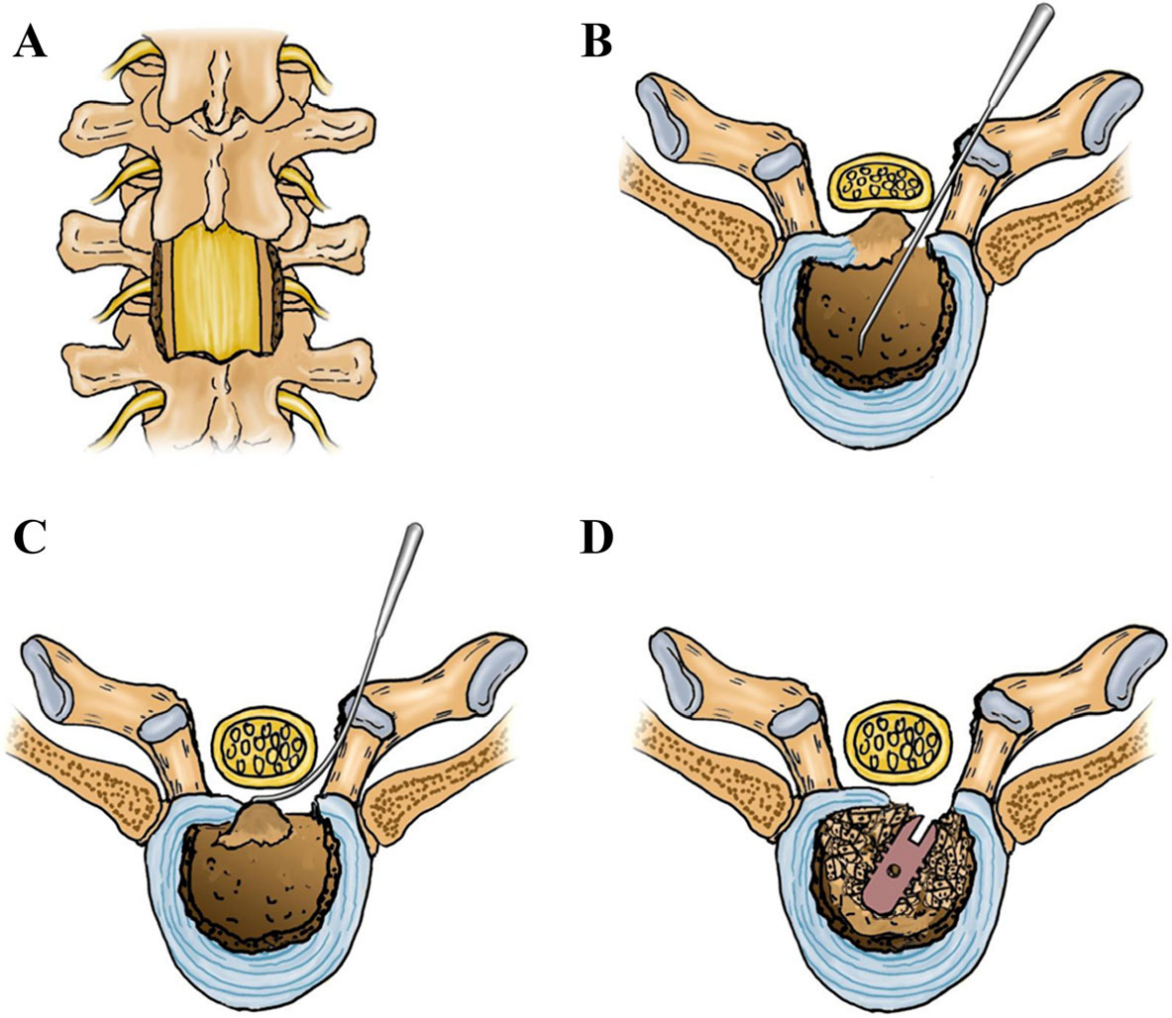
**a** The spinous process and lamina and bilateral articular process in affected segment were removed so as to expose the dural sac and intervertebral disc. **b** The intervertebral space was hollowed out to form an “eggshell,” after the removal of disc tissue within the intervertebral space. **c** The adhesion between the herniated calcified disc and dura mater was separated by nerve retractor crook or hook knife, and the hardening disc tissue was pushed to the anterior intervertebral space by the reverse angled curette. **d** Autologous bone graft and a cage were implanted into the interbody for fusion purpose. The pedicle screw-rod system was fixed in situ in order to prevent a slight curvature change after decompression, thereby reducing damage to the spinal cord

**Fig. 2:**
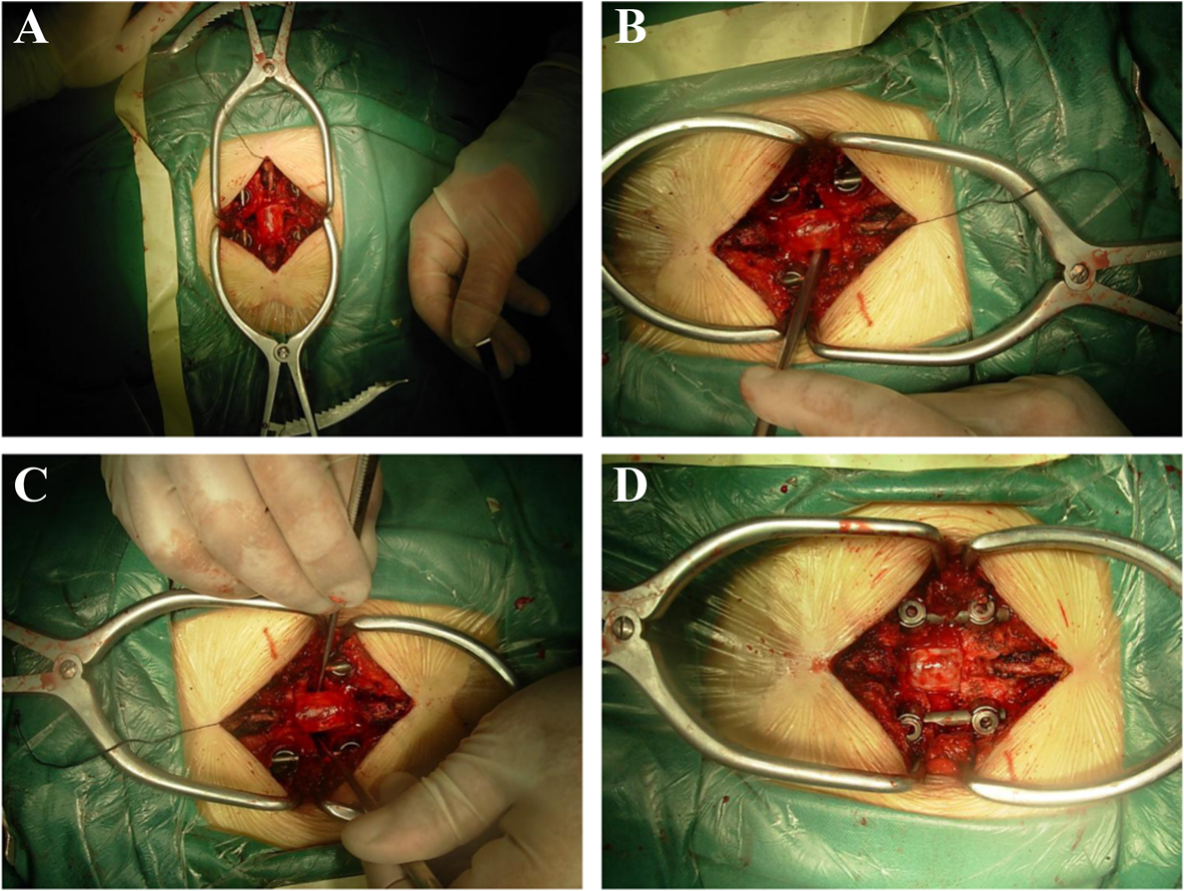
**a** The spinous process and lamina and bilateral articular process in affected segment were removed so as to expose the dural sac and intervertebral disc. **b** The intervertebral space was hollowed out to form an “eggshell,” after the removal of disc tissue within the intervertebral space. **c** The adhesion between the herniated calcified disc and dura mater was separated by a nerve retractor crook or hook knife, and the hardening disc tissue was pushed to the anterior intervertebral space by the reverse angled curette. **d** Autologous bone graft and a cage were implanted into the interbody for fusion purpose. The pedicle screw-rod system was fixed in situ in order to prevent a slight curvature change after decompression, thereby reducing damage to the spinal cord

The patient was under general anesthesia by endotracheal intubation in the prone position. After positioning by C-arm, a 10-cm-long posterior midline incision was made in the level of the affected area. The spinous process, bilateral upper and lower vertebral transverse process, and articular process were exposed, without operation on the intercostal nerve, arteries, and veins. The pedicle screws were inserted into the decompression segment of the vertebral body. The results of intraoperative fluoroscopy indicated that the positions were good. The spinous process and lamina were removed by uncover method. The operator should be cautious while uncovering the lamina. Once encountering resistance or the traction of the dura mater, we should check that there was dura mater adhesion or not so as to prevent spinal cord injury. The adhesion site could be separated by a nerve retractor. If it was hard to separate the adhesion, partial lamina could be preserved in the dura matter. The bilateral facet joints were removed so as to expose fully the spinal cord and dura matter (Figs. 1a and 2a). The herniation location, extent, and adhesion degree with the dura matter were confirmed.

**Fig. 3:**
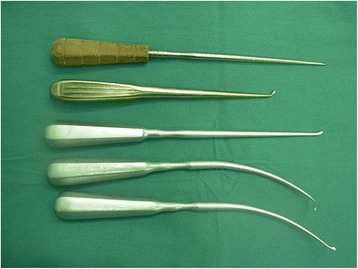
The hooks, straight reverse curette, and angled reverse curette used in an eggshell procedure

After removal of the vertebral body corner by a clamp, the annular fibrosus was cut open. The disc tissue within the intervertebral space was removed, leaving only the front and part of the side of the annulus (Figs. 1b and 2b). The adhesion between the herniated calcified disc and dura mater was separated by a nerve retractor crook or hook knife, and the hardening disc tissue was pushed to the anterior intervertebral space by the reverse angled curette (Figs. 1c and 2c). This was the “modified eggshell prcedure”. The special equipments were shown in Fig. 3. The hardening disc tissue was removed by the curved clamp. If the adhesion between ossification tissue and spinal dura mater was close, the ossified tissue could be abrasively reduced so as to be free from surrounding tissues, making it “floated”. The removed bone of spinous process and lamina and articular process in combination with a cage (or vertically implanting a cage in each side) were inserted into the intervertebral space. The pedicle screw-rod system was fixed in situ in order to prevent a slight curvature change after decompression, thereby reducing damage to the spinal cord (Figs. 1d and 2d). After complete hemostasis and drainage set, the incision was sutured layer by layer. All patients were preventively treated with antibiotics for 3 days after operation and postoperatively stayed on bed for 1 week. Then, the patients could get out of bed with waist protection.

### The postoperative follow-up and therapeutic effect assessment

The clinical follow-up observation was carried out by outpatient review. The patients were followed up rigorously by independent evaluators at 3, 6, and 12 months after operation. After then, the patients were followed up once every year. The last follow-up result was used as postoperation evaluation index. JOA score was used to evaluate the spinal cord function improvement. Except for the upper limb function, the rest of the total score was 11 points. Based on the preoperative, postoperative, and last follow-up score, the improvement rate was calculated. According to the improvement rate, the clinical effect was divided into four grades: improvement rate ≥50 %, good; 10 % ≤ improvement rate < 50 %, moderate; 0 % ≤ improvement rate < 10 %, no improvement; improvement rate <0 %, deteriorated [10]. During the follow-up period, X-ray image and three-dimensional CT scan were taken to observe the fusion situation according to the method reported by Brantigan and Steffee [11].

### Statistical analysis

SPSS18.0 statistical software (SPSS Inc., USA) was used for data analyses in this study. All data were presented as mean ± standard deviation (SD) or median (IQR, interquartile range) depending on the normal or abnormal distribution of the data. JOA score preoperatively and that at follow-ups after operation were compared using repeated measurement analysis of variance (ANOVA), accompanied by Mann-Whitney *U* test for pairwise comparison. A *p* value less than 0.05 was considered as statistically significant.

## Results

The operations were successfully completed. For four cases in this study, the spinal cord-floating method was adopted, because the adhesion between ossified tissue and dural mater was very close. The operation time ranged from 150 to 310 min, with an average of 222 min. The amount of bleeding was 400~1100 ml with an average of 722 ml. Twenty-one patients received blood transfusion intraoperatively, with median transfusion of 600 ml.

There were two cases of dural laceration. There was ligamentum flavum ossification in one case of them. The epidural dorsal was teared in that case while separating the ossified ligamentum flavum from epidural adhesions. And then the tear was sutured by 5-0 non-invasive sutures. There were disc calcification and epidural adhesions in the other case. The ventral epidural dorsal was teared while separating the adhesion. The tear was repaired with artificial dural patch and covered by gelatin sponge. For the above two cases, the muscle and fascia tissue were sutured closely. The postoperative drainage was persistent, and the wound was bandaged. After absolute bed rest for 1 week with extension of extubation (7 days), the incisions were healed with no significant sequelae.

For one case in this study, the neural symptoms of lower extremity got worse at time of 8 h after operation. The emergency MRI showed that there was spindle abnormal signal in the left rear part of spinal canal, presenting short T1 and long T2 mixed signal. The boundary was clear, and the corresponding spinal structure was under pressure. The preliminary impression was subdural hematoma. After an emergency operation performed, it was found that the neural symptom was caused by poor drainage due to the distortion of drainage tube. After removal of 200 ml non-condensable blood, with the use of hormones, dehydration, and neurotrophic medications, the symptoms of this patient were improved rapidly. The muscle strength recovered to the preoperative level 48 h after operation.

All of the patients were followed up for 5 to 8 years, with the median of 6 years. The neural function of the patients achieved different degrees of recovery. JOA score of preoperation was 5 (IQR = 1) while it was 8 (IQR = 2) at final follow-up, with significant difference (Mann-Whitney *U* test, *Z* = −4.891, *P* < 0.001). The improvement rate of neurological status was 51.5 ± 23.1 %. According to the classification of improvement rate, there were 15 cases at good level, 8 cases at moderate level, 2 cases without any improvement, and no cases with deterioration. For the 2 cases without improvement, the preoperative JOA score was only 2 points in both of them. The severe depression and denaturation had already existed preoperatively. Although the oppressed tissue was removed completely in the process of operation, the spinal function did not recover. The JOA score was still only 2 points at last follow-up after operation.

**Fig. 4:**
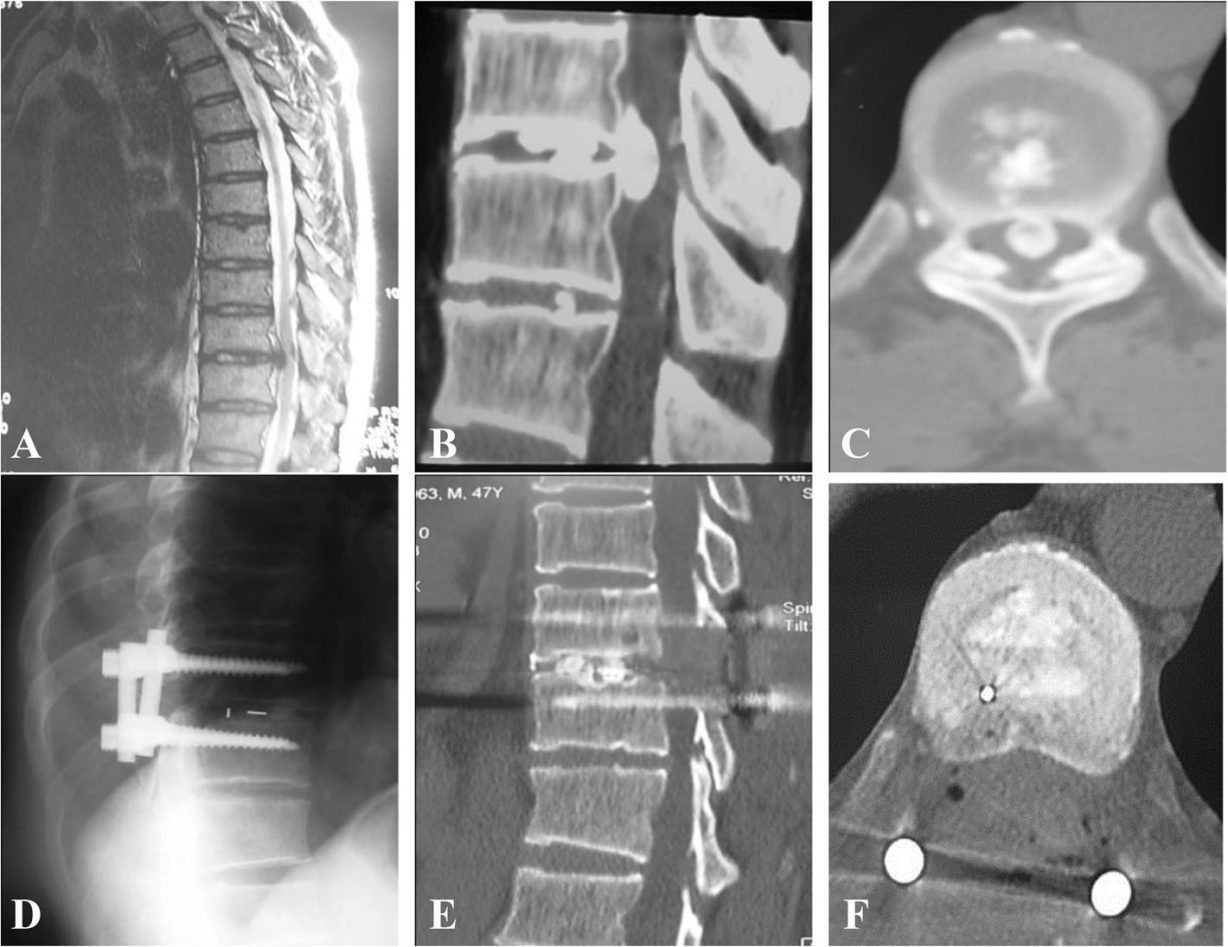
Representative medical images for eggshell procedure. **a** Preoperative MRI scan showed that there was disc herniation in T9/10 level, and the dura mater was oppressed. **b** Preoperative sagittal CT scan showed that there was T9/10 disc herniation with calcification. **c** Preoperative axial CT scan showed that there was T9/10 disc herniation associated with calcification. **d** Postoperative X-ray showed that the internal fixation was good, and there was intervertebral bone grafting. **e**, **f** The postoperative sagittal and axial CT scan showed that the sclerosing herniated disc was removed completely with decompression at 360°

According to the observation method of bone graft fusion described by Brantigan and Steffee [11], 3 months after operation, 23 patients reached grade E fusion (firm fusion). The bone density in the fusion region was mature and dense, and the mature bone trabeculae were formed into bone bridges. Two other patients reached grade D fusion (suspicious fusion). The bone bridges in the fusion region were formed, and their density were at least equal to the postoperative state. Twelve months after operation, all of the patients reached grade E fusion. There was no fixation loosening, fracture, and other complications in the last follow-up (Fig. 4). None of thoracic kyphosis and local instability signs occurred.

## Discussion

### The clinical features and surgical approach of sclerosing thoracic disc herniation

The incidence of thoracic disc herniation was low, and the clinical manifestations were not typical. The diagnosis was easily delayed. The classification of thoracic disc herniation was determined by the segment and location of herniation. The classification was helpful for surgical method selection. According to the herniation parts, the thoracic disc herniation was divided into central type, paracentral type, lateral type, and intradural type. The main symptoms of central herniation type were spinal cord damage, and the main symptoms of lateral herniation type were radicular symptoms. The patients of intradural type were rare. The thoracic disc herniation was often accompanied with calcification and posterior marginal osteophyte formation, known as the rigid herniation [2]. In early time, it was reported [12] that 65 % of 82 cases of thoracic disc herniation occurred with intervertebral disc calcification and 7 % of 82 cases with intradural herniation. Hott et al. [13] reported that in a group (20 cases) of huge thoracic disc patients, there were 18 cases of rigid herniation, 1 case of soft herniation and 1 case of partial calcification.

The etiology and pathology mechanism of hardening thoracic disc herniation was not completely clear. The related imaging studies showed that the hardening thoracic disc herniation was often accompanied with ossification of posterior longitudinal ligament, yellow ligament ossification, the posterior edge transection and wedging of vertebral body, and so on. The clinical manifestations of hardening thoracic disc herniation were often severe. The herniation and hardening part usually deepened into the ventral spinal dural with adhesions, even causing mechanical compression on thoracic spinal cord and influencing spinal cord blood by affecting the anterior spinal central artery. Once the symptomatic thoracic disc herniation was confirmed, the patient had better receive surgical treatment as soon as possible [6]. The surgery indication [7, 8] included progressive spinal cord damage, lower limb weakness or paralysis, bowel and bladder dysfunction, segmental sensory disturbances, and persistent radiating pain.

There were several surgical methods to treat thoracic disc herniation [14], and the surgical procedures of posterior laminectomy, discectomy, and decompression are relatively simple. However, the intraoperative traction to the affected spinal cord was easy to cause spinal cord injury. Even the minor damage to the spinal cord can also cause irreversible damage of the nerve function, influencing the further functional recovery. Moreover, the surgical operation was difficult to perform because the affected spinal cord was under the inflammation, ischemia, and decompensated status for a long time, and the surgical risk was high [4].

Regarding surgical techniques for ossification of posterior longitudinal ligament (OPLL) in the thoracic spine, Prof. Kato and Murakami from Japan have done a lot of research and contributed much. In the year 2012, they firstly reported a novel surgical technique for OPLL in the thoracic spine [15]. Their maneuver included laminectomy and removal of the transverse processes and pedicles, which allowed space to be created bilaterally at the sides of the dural sac for the subsequent anterior decompression. The thoracic nerves at the levels of anterior decompression were ligated bilaterally and lifted up to manipulate the ossified ligament and the dural sac. An anterior decompression was then performed posteriorly. Complete removal of the ossified PLL was achieved in all cases. Recently, they reported that it was safe and effective for gradual spinal cord decompression through migration of floated plaques after anterior decompression via a posterolateral approach for OPLL in the thoracic spine [16]. Besides, they reported in another study [17] a case of severe anterior compression of the spinal cord by a calcified herniated thoracic disc at the T9/10 level in a 46-year-old woman. They performed resection of the calcified herniated thoracic disc and the integrated dura, using a microscopically assisted mini-open transthoracic approach in which the rib bone was partially drilled to widen the operative field, which was a successful attempt. The patient achieved an excellent recovery without any complications. Obviously, our modified eggshell procedure is different from what they reported before. However, the postoperative results are satisfying although we have used different methods.

The front side transthoracic approach for disc resection was used widely. In such procedure, the surgical field was very clear and direct, and the ventral spinal cord decompression had less interference to the spinal cord. However, the possibility of postoperative pulmonary complications increased significantly. The clinical effect was good to treat thoracic disc herniation by posterolateral approach through articular process, with shorter operative time and fewer complications in comparison with other treatment methods [18]. To our knowledge, eggshell procedure was firstly used for anterior decompression of spinal fractures via a posterior way and then used widely [9]. As eggshell procedure, vertebral body is firstly hollowed out via vertebral pedicle, and then the protruding fracture block is rammed into the hollowed vertebral body. In this study, we modified this procedure and applied it to the surgical treatment of sclerosing thoracic disc herniation. On the basis of the advantages mentioned above, we used the modified eggshell procedure, obviously decreasing the risk of damaging the spinal cord. In this study, the modified eggshell procedure had small interference to the spinal cord. No cases were found with postoperative neurological deterioration due to spinal cord traction.

### The key points of operation and advantages of modified eggshell procedure through posterior approach

The eggshell procedure was firstly used for spinal fracture with anterior decompression. The vertebral body was hollowed out through the pedicle, and the kyphotic fracture bones were inserted into the vertebral body. At present, the eggshell procedure was mainly used in spinal deformity correction. We modified this procedure and used it in the surgical treatment of hardening thoracic disc herniation. The articular process was removed so as to expose the side and rear side of intervertebral space through posterior approach. The affected disc tissue which was broken into the spinal canal was removed from the intervertebral space so as to alleviate the spinal compression through the anterior approach. Such procedure realized decompression through the anterior spinal cord by single posterior surgical operation with small disturbance for the spinal cord, and the operation was relatively safe.

The key points of modified eggshell procedure were as follows. First, the laminectomy and bilateral facet were removed to expose the anterolateral aspect of affected intervertebral space. Second, the osteophytes in posterior margin of vertebral body and intervertebral disc tissue were removed through the anterolateral aspect of the intervertebral space. Third, the adhesion between the herniated calcified disc and dura mater was separated in the anterior aspect of the dura mater. The nucleus pulposus pliers were used to completely decompress in the intervertebral space. If the adhesion between the ossified tissue and spinal dura mater was very close, the ossified tissue could be abrasively reduced so as to be free with surrounding tissue, making it “float,” thus effectively avoiding dura mater damage and reducing the occurrence of cerebrospinal fluid leakage.

The advantages of this procedure were as follows. First, the posterior surgery alone could reach full laminectomy, and remove the hypertrophy ligamentum flavum, bilateral facet joint and affected disc. Videlicet, it could completely alleviate the spinal stenosis, thus achieving 360° decompression. Second, the spinal disc which was broken into vertebral canal was removed from the ventral spinal cord. The traction on the dura mater should be avoided in order to decrease the risk of damaging the spinal cord, especially when there were difficulties in the anterior removal process due to the adhesion between the herniation disc and dura mater. Third, there was no important anatomical structure in the posterior section. The operation in this way was easy and the damage was less, generally with fewer incision-related serious complications. The transthoracic surgical approach should not be selected so as to avoid pulmonary complications and major vascular injury. The previous study [19] indicated that the lung function after thoracotomy at least decreased 10 %. Because of the obstruction by the diaphragm in the level of T10/L1, it was difficult to expose the structure through the anterior approach and was prone to complications such as pleural effusion [20]. Fourth, all of the surgical operations were under direct vision. There was enough space to remove the compressed tissue in the anterior approach, and the operation was easy and safe. Barbanera et al. [21] reported that the direct vision for the dura mater was very important in the process of operation while dealing with a huge thoracic disc herniation, especially with calcification. Unavoidably, it goes along with some limitations in this study. One limitation is the retrospective study design, with some medical records lost. Another limitation is lack of control group. That is, it would be better if a comparative study is performed, which is needed in the future.

The complication rate is 12 % in this study, including two cases of dural laceration and one subdural hematoma, all transient. Takahata et al. [22] reported a relatively high rate of surgical complications, which included a dural tear in 40 % and neurological deterioration in 33 % of the patients. Kato et al. [15] reported a minor dural tear in one of three patients that did not result in postoperative cerebrospinal fluid leakage. In another study from Kato et al. [16], two patients had operative complications (six cases in total), including a dural tear and a temporary neurological deterioration. Thus, our 12 % complication rate is relatively low, as compared to that reported before.

### The necessity of interbody fusion and internal fixation

This surgical method removed the spinal posterior structure in operation session, influencing the spinal stability and leading to local curvature change. Especially for the lower thoracic, it was prone to instability and curvature changes due to the weakened thorax protection. Furthermore, after the removal of the disc, the anterior and middle column damage might worsen the instability of spine. Because of long-term compression, the spinal cord was rather fragile. Therefore, even a slight change of curvature might also cause serious damage to the spinal cord. The biomechanical study [23] indicated that after the removal of unilateral thoracic rib vertebral joints, transverse process joint of ribs or unilateral facet joints and bilateral facet joints, the local stability decreased. The anterior and middle column beared 80 % of the load, while the posterior column only beared 20 % of the load. The interbody fusion might provide a reliable support for spine. Therefore, we adopted the interbody fusion and pedicle screw with rod system which was fixed in situ fusion in order to enhance the stability of operating sessions, effectively avoiding the slight curvative changes after decompression, thereby reducing damages to the spinal cord. The application of autologous lamina and small joint bone for interbody fusion [24] could avoid the complications of iliac area removal and reduce the surgery time, reducing operative bleeding. According to the current follow-up data, the integration and stability effect was reliable, and the spinal damage caused by bone defluxion did not occur. The application of internal fixation was helpful for early ambulation of the patients to do functional exercise and helpful for providing support to the interbody fusion, effectively reducing postoperative bedridden complications, shortening recovery time, and improving the quality of life for patients.

## Conclusions

In summary, modified eggshell procedure via posterior approach is a safe and effective surgical method when performed to treat sclerosing thoracic disc herniation in the clinical practice.

## Abbreviations

ANOVA: Analysis of variance
CT: Computed tomography
IQR: Interquartile range
JOA: Japanese Orthopaedic Association
MRI: Magnetic resonance imaging
SD: Standard deviation
T1WI: T1-weighted imaging
T2WI: T2-weighted imaging
